# Characterizing genetic interactions in human disease association studies using statistical epistasis networks

**DOI:** 10.1186/1471-2105-12-364

**Published:** 2011-09-12

**Authors:** Ting Hu, Nicholas A Sinnott-Armstrong, Jeff W Kiralis, Angeline S Andrew, Margaret R Karagas, Jason H Moore

**Affiliations:** 1 Department of Genetics, Dartmouth Medical School, Dartmouth College, Lebanon, NH, USA; 2 Department of Community and Family Medicine, Dartmouth Medical School, Dartmouth College, Lebanon, NH, USA; 3 Institute for Quantitative Biomedical Sciences, Dartmouth Medical School, Dartmouth College, Lebanon, NH, USA

## Abstract

**Background:**

Epistasis is recognized ubiquitous in the genetic architecture of complex traits such as disease susceptibility. Experimental studies in model organisms have revealed extensive evidence of biological interactions among genes. Meanwhile, statistical and computational studies in human populations have suggested non-additive effects of genetic variation on complex traits. Although these studies form a baseline for understanding the genetic architecture of complex traits, to date they have only considered interactions among a small number of genetic variants. Our goal here is to use network science to determine the extent to which non-additive interactions exist beyond small subsets of genetic variants. We infer statistical epistasis networks to characterize the global space of pairwise interactions among approximately 1500 Single Nucleotide Polymorphisms (SNPs) spanning nearly 500 cancer susceptibility genes in a large population-based study of bladder cancer.

**Results:**

The statistical epistasis network was built by linking pairs of SNPs if their pairwise interactions were stronger than a systematically derived threshold. Its topology clearly differentiated this real-data network from networks obtained from permutations of the same data under the null hypothesis that no association exists between genotype and phenotype. The network had a significantly higher number of hub SNPs and, interestingly, these hub SNPs were not necessarily with high main effects. The network had a largest connected component of 39 SNPs that was absent in any other permuted-data networks. In addition, the vertex degrees of this network were distinctively found following an approximate power-law distribution and its topology appeared scale-free.

**Conclusions:**

In contrast to many existing techniques focusing on high main-effect SNPs or models of several interacting SNPs, our network approach characterized a global picture of gene-gene interactions in a population-based genetic data. The network was built using pairwise interactions, and its distinctive network topology and large connected components indicated joint effects in a large set of SNPs. Our observations suggested that this particular statistical epistasis network captured important features of the genetic architecture of bladder cancer that have not been described previously.

## Background

Identifying associations between genetic and phenotypic variation is crucial to understanding the genetic basis of disease susceptibility and disease etiology [1], and to devising diagnostic tests and useful treatments [2,3]. With the rapid expansion of open-access single nucleotide polymorphism (SNP) databases [4], the progress in genotyping technologies [5], and the availability of immense computational resources [6], mapping the genes that underlie common diseases and quantitative traits is now feasible.

Genome-wide associations studies (GWAS), in which thousands to millions of SNPs across the human genome are tested for associations with disease phenotypes, have emerged as a particularly promising approach for drawing causal inferences between traits and genetic variation [2,3,7,8]. However, although GWAS have uncovered numerous disease susceptibility loci [3,8,9], the majority of them have had relatively subtle individual associations with disease risk. The success of GWAS analyzed only for individual SNP effects largely depends on fundamental assumptions about a lack of genetic complexity and a simple single-gene architecture of diseases, and becomes greatly compromised when gene-environment or gene-gene interactions modify the relationship between genotypes and phenotypes [10-13].

The genetic architecture of common human diseases is, in fact, characterized in part by interactions between genes, i.e., *epistasis *[13-19]. Accordingly, the focus of recent research has shifted from identifying single locus susceptibility [2,7] to quantifying interaction effects between multiple candidate loci throughout the human genome [13,16,20,21]. However, the study of epistasis faces an initial challenge arising from the existence of fundamental differences between the concepts of biological and statistical interaction (e.g. [21]). These differences imply that *statistical epistasis*, defined at the population level as the non-additive mathematical relationship among multiple genetic variants, cannot be literally translated into *biological epistasis*, which is the physical interaction among two or more molecules at the cellular level of an organism, and vice-versa [17]. Moreover, detecting gene-gene interactions and accounting for them in GWAS further represents a statistical and computational challenge [12,13,20,22]. The statistical challenge results from the prohibitive amount of data necessary to support the huge number of hypotheses involved in modeling interactions, even when considering only pairwise interactions [3,11]. The computational challenge, in turn, arises from the necessity to exhaustively evaluate all possible combinations of SNPs, which becomes infeasible when interactions involve more than two SNPs: the computational complexity, which is in the quadratic order for pairwise interactions, increases exponentially with higher-order interactions, rendering any exhaustive assessment impossible [12,13,21].

The necessity to overcome these difficulties calls for efficient tools to detect genetic interactions [2,7,23]. Methods such as machine learning [24-26] and dimensionality reduction [27,28] have recently proven useful in detecting influential interactions. However, these approaches are aimed at identifying best models consisting of several SNPs and thus ignore the broader gene-gene interaction landscape.

A particularly intuitive approach to explore the genetic architecture of common human diseases and to identify genetic interactions is to use networks. A network is generally defined as a collection of vertices joined in pairs by edges and is a powerful tool to represent and study complex systems [29,30]. In biological systems, for instance, networks can be used to characterize interactions at all levels of organization, from the molecular level with metabolic [31,32], protein-protein interaction [33], and genetic regulatory networks [34], to the macroscopic level with food webs [35].

Networks allow for a structured representation of a collection of entities and their relationships, which provides a well-suited framework for the study of epistasis. The use of networks does not resolve the dimensionality problems inherent in exploring high-order interactions amongst multiple SNPs. An intuitive solution that has previously proven helpful is to filter out the considerable noise masking the useful genotypes and to reduce the search space to a subset of high-susceptibility SNPs before constructing a network of genetic interactions.

An example of such a sequential approach is the work of McKinney et al. [36], who developed a genetic-association interaction network to visualize and interpret synergetic interactions between pairs of SNPs. Loci were initially chosen based on the strength of their main effects. Although useful, purging databases for irrelevant genetic variants and preliminarily selecting high-susceptibility SNPs inevitably comes at the risk of discarding loci comprised in significant higher order interactions. Hence, alternative solutions for reducing the space of possible interactions in GWAS are needed.

In the present study, we propose to infer genetic interaction networks that are not dependent on statistical main effects. We first rank all possible pairwise interactions between SNPs according to their relative strength and subsequently build and analyze *statistical epistasis networks *including only those interactions whose strength exceeds a given threshold. Hence, the approach we apply distinguishes itself from existing ones in the following ways: 1) We qualify the strength of all pairwise interactions identifiable in the complete data set rather than a subset of high main-effect SNPs; 2) We organize our genetic network around the strongest pairwise interactions rather than around the strongest main effects; 3) We analyze network topologies to systematically identify the network that best captures the genetic architecture inherent in the data; 4) In contrast to many existing techniques that aim at identifying a classification model consisting of a subset of susceptibility SNPs, our epistasis network captures a broader landscape of gene-gene interactions through exhaustively enumerating all possible pairwise interactions.

In the United States, bladder cancer is one of the most common types of cancer in both men and women. Although the main known cause of bladder cancer is smoking [37], recent case-control studies also suggest that there exist heritable susceptibility factors [38-40]. Thus, we used the network approach to characterize the space of pairwise interactions in a bladder cancer data set consisting of 1,422 SNPs sampled across 491 patients newly diagnosed bladder cancer and 791 controls [41]. Statistical epistasis networks were built by incrementally adding edges between SNPs if the strength of their pairwise interactions was greater than a given threshold. We identified one threshold value for which the resulting network showed unique topological characteristics, which we believe, capture the complex structure intrinsic in the data. Its distinctively large connected component suggests that a group of SNPs may jointly modify the disease outcome. Thus, the network may serve as a scaffold to explore the landscape of higher-order interactions.

## Methods

### Bladder cancer data set

The data set used in this study consisted of cases of bladder cancer among New Hampshire residents, ages 25 to 74 years, diagnosed from July 1, 1994 to June 30, 2001 and registered in the State Cancer Registry. All controls were selected from population lists. Controls less than 65 years of age were selected using population lists obtained from the New Hampshire Department of Transportation, while controls aged 65 and older were chosen from data files provided by the Centers for Medicare & Medicaid Services (CMS) of New Hampshire. This data set also shared a control group with a study of non-melanoma skin cancer in New Hampshire covering an overlapping diagnostic period of July 1, 1993 to June 30, 1995 and July 1, 1997 to March 30, 2000. Additional controls were selected for bladder cancer cases diagnosed in the intervening period frequency matched to these cases on age (25-34, 35-44, 45-54, 55-64, 65-69, 70-74 years) and gender.

Informed consent was obtained from each participant and all procedures and study materials were approved by the Committee for the Protection of Human Subjects at Dartmouth College. Consenting participants underwent a detailed in-person interview, usually at their homes. Recruitment procedures for both the shared controls from the non-melanoma skin cancer study and additional controls were identical and ongoing concomitantly with the case interviews.

DNA was isolated from peripheral circulating blood lymphocyte specimens harvested at the time of interview using Qiagen genomic DNA extraction kits (QIAGEN Inc., Valencia, CA). Genotyping was performed on all DNA samples of sufficient concentration, using the GoldenGate Assay system by Illumina's Custom Genetic Analysis service (Illumina, Inc., San Diego, CA). Out of the submitted samples, 99.5% were successfully genotyped and samples repeated on multiple plates yielded the same call for 99.9% of SNPs. The missing genotypes were imputed using a frequency-based method. That is, the missing value of an individual was filled using the most common genotype of the corresponding SNP in the population. The data set used in our analysis consisted of 491 bladder cancer cases and 791 controls and most (> 95%) of the subjects were of Caucasian origin. More details on this data set and the methods are available in [40,41].

### Network construction

Networks are formalized mathematically by graphs, and we use these two terms interchangeably in this article. A graph *G *is composed of a set *V *(*G*) of vertices and a set *E*(*G*) of edges [42]. In our epistasis networks, each vertex corresponds to a SNP, and we use *v_A _*to denote the vertex corresponding to SNP *A*. An edge linking a pair of vertices, for instance *v_A _*and *v_B_*, corresponds to an interaction between SNPs *A *and *B*.

We first assigned a weight to each SNP and each pair of SNPs to quantify how much of the disease status the corresponding SNP and SNP pair genotypes explain. In analogy to statistical models, those weights correspond to the strength of the main and the interaction effects and stronger effects translate into higher weights. In information theoretic terms, those weights correspond to the so-called *mutual information *and *information gain *[43]. Specifically, the weight of SNP *A *is *I*(*A*; *C*), the mutual information of SNP *A*'s genotype and *C*, the class variable with status *case *or *control*. Intuitively, *I*(*A*; *C*) is the reduction in the uncertainty of the class *C *due to knowledge about SNP *A*'s genotype. Its precise definition is

(1) I(A;C)=H(C)-H(C|A),

where *H*(*C*) is the *entropy *of *C*, i.e., the measure of the uncertainty of class *C*, and *H*(*C*|*A*) is the *conditional entropy *of *C *given knowledge of SNP *A*. Entropy and conditional entropy are defined by

(2) $$H\left(C\right)=\underset{c}{\sum }p\left(c\right)\log\frac{1}{p\left(c\right)},$$

(3) $$H\left(C|A\right)=\underset{a,c}{\sum }p\left(a,c\right)\log\frac{1}{p\left(c\left|a\right)\right)},$$

where *p*(*c*) is the probability that an individual has class *c*, *p*(*a*, *c*) is that of having genotype a and class *c*, and *p*(*c*|*a*) is that of having class *c *given the occurrence of genotype *a*. In our implementation, *p*(*c*) is the frequency of diseased (case) or healthy (control) individuals respectively, *p*(*a*, *c*) is the frequency of individuals in either the case or the control group that carry genotype *a*, and *p*(*c*|*a*) = *p*(*a*, *c*)/*p*(*a*), where *p*(*a*) is the frequency of individuals that have genotype *a*. Given that in most cases a SNP has two alleles and there are consequently three possible genotypes for each SNP in the diploid human genome, the sum in equation (3) is over all six possible combinations of genotypes *a *and classes *c*. Mutual information *I*(*A*; *C*) takes only non-negative values. If the class *C *is independent of a SNP *A*'s genotype, *I*(*A*; *C*) = 0, i.e., SNP A does not predict the disease status. If a correlation exists between the class *C *and SNP *A*, *I*(*A*; *C*) > 0, i.e., SNP *A *has a main effect and predicts some of the disease status. Larger values of *I*(*A*; *C*) indicate stronger correlations between *A *and *C*.

Given the pair of vertices *v_A _*and *v_B_*, its weight is the information gain *IG*(*A*; *B*; *C*), where

(4) IG(A;B;C)=I(A,B;C)-I(A;C)-I(B;C).

As such, *IG*(*A*; *B*; *C*) is the reduction in the uncertainty, or the information gained, about the class *C *from the genotypes of SNPs *A *and *B *considered together minus that from each of these SNPs considered separately. In brief, *IG*(*A*; *B*; *C*) measures the amount of synergetic influence SNPs *A *and *B *have on class *C*. A higher value indicates a stronger synergetic interaction. Note that *IG*(*A*; *B*; *C*) can take non-positive values. A negative value indicates that the genotypes of two SNPs tend to vary together (redundant information), while a value of zero indicates either that the genotypes of the two SNPs are independent or, more likely, that they interact with a mixture of synergy and redundancy. The synergetic part of the mix tends to make the information gain positive while the redundant part lowers the information gain.

Information theory has previously been applied in epistasis studies. For instance, Moore et al. [44,45] used interaction dendrograms based on information gain to interpret their epistasis models. McKinney et al. [36] used information gain to quantify synergic interactions between pairs of SNP in their genetic-association interaction network. In a more general framework, Jakulin and Bratko [46] used mutual information and information gain to quantify the information shared by single class variables and their corresponding attributes. Although there are many other approaches, such as MDR, random forest, and logistic regression, that are able to measure the strength of main and interaction effects of SNPs, we specifically chose information theoretical measures in this study because they are more computationally efficient than the others. This is very important in the era of GWAS since inferring interactions on a genome-wide scale is very computationally intensive.

We then built a series of statistical epistasis networks by incrementally adding edges. These networks were denoted by *G_t_*, where edges between SNPs were added only if their pair weights were greater than or equal to a threshold *t*. The threshold *t *varied between 0 and the maximum pair weight estimated based on the data. The networks *G_t _*grew as the threshold *t *decreased. For *t*_1 _<*t*_2_, $G_{t_{1}}$ contained all the edges and vertices of $G_{t_{2}}$.

### Network analysis

Our analysis method relies on comparisons between the real data set and its derivatives generated by permutation testing. First, permuted data were used to assess the significance level of the interaction strength of each SNP pair. Second, and more importantly, by comparing networks built from real data and permuted data, we can determine the statistical significance of the network properties themselves. We repeated the network construction and characterization exactly the same way on both real data and permuted data. Thus, any network features observed in the real data that were not consistent with the distribution of features from the permuted data can be inferred to be due to real genetic associations.

We generated 1,000 permuted data sets by randomly shuffling the disease status of the 1,282 samples 1,000 times. This removed all biological signals from the data. For each permuted data set, we then calculated the weights for all pairs of SNPs and constructed a series of networks using the same thresholds as when we built the real-data networks. Once all the networks were assembled, we first evaluated the significance of each pair of SNPs in the real data set by calculating the fraction of permuted data sets with pair weight greater than that obtained from the real data. Then, we investigated and compared some basic properties of these series of networks.

The four basic properties of a network considered here are the number of edges, the number of vertices, the size of the largest connected component, and the vertex degree distribution. The definitions of these standard graph-theoretic terms [42] are summarized as follows. A *connected component *of a graph is a maximal connected subgraph, and the size of a connected component refers to its number of vertices. A graph *H *is a subgraph of *G *if both the vertex set and edge set of *H *are subsets of those of *G*. A subgraph is *connected *if any two vertices in it can be joined by a sequence of edges. The *degree *of a vertex *v*, denoted by *d*(*v*), is the number of edges incident with *v*. The fraction of vertices in a network that have degree *d *is denoted by *p*(*d*). Thus, *p*(*d*) can be viewed as the probability that a randomly chosen vertex in the network has degree *d*. The quantities *p*(*d*) make up the *vertex degree distribution *of a network. In the context of epistasis networks, the degree of vertex *v_A _*indicates how many SNPs interact with SNP *A*, while the clustering of vertices within a connected component may help narrow the search for informative SNPs likely to jointly modify disease outcome.

## Results

### Measures of main and interaction effects in the bladder cancer data

**Figure 1:**
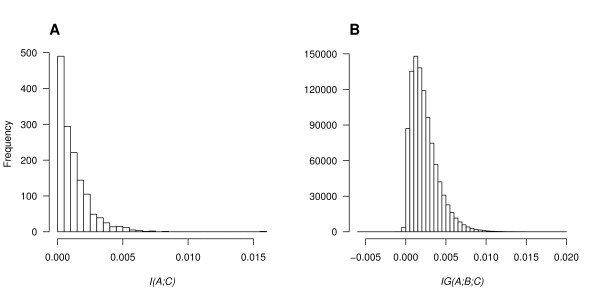
**Frequency distributions of the mutual information and the information gain from the real data set**. **A **Frequency distribution of main effects for all 1,422 SNPs. The values of *I*(*A*; *C*) range from 0 to 0.01551. **B **Frequency distribution of pairwise interactions for all 1,010,331 pairs of SNPs. The values of *IG*(*A*; *B*; *C*) range from -0.00591 to 0.01967.

As shown in Figure 1-A, most of the 1,422 SNPs had relatively small main effects (*mean *± *stdev *= 0.00122 ± 0.00125) and a few SNPs had very strong main effects. The highest weight was 0.01551 for SNP *IGF2AS*_*04 *and the second highest weight, which was about half of the highest, was 0.00832 for *LRP5*_*12*. The distribution of interaction strengths (Figure 1-B) had *mean *± *stdev *= 0.00235 ± 0.00171. The highest weight was 0.01967, and corresponded to the interaction between SNPs *ESR2*_*02 *and *TERT*_*25*. Of all $\left(\begin{array}{c} 1422\\ 2 \end{array}\right)=1,010,331$ pairs of SNPs, there were 778 pairs with a weight of zero, and 3,083 with negative weights.

### Network investigations

The four topological features of *G_t _*and of the permuted-data networks were investigated. All these features were found to distinguish the structure of *G_t _*from the permuted-data networks. The network *G*_0.013 _was of special interest by showing the most significant network topologies, and is considered in some detail at the end of this section.

#### Numbers of edges and vertices

**Figure 2:**
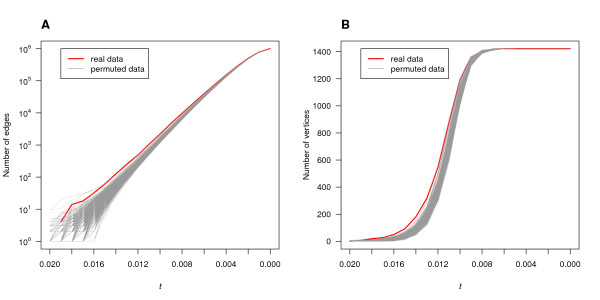
**Network growth with decreasing threshold *t***. **A **Increase in the number of edges. **B **Increase in the number of vertices. In both graphs, the red line represents *G_t _*of the real data and the gray lines represent networks of 1,000 permuted data sets. The threshold *t *decreases from 0.02 to 0 in increments of 0.001.

Recall that the existence of an edge linking SNPs *A *and *B *in the epistasis network *G_t _*indicates an interaction of strength *IG*(*A*; *B*; *C*) ≥ *t *between them and the networks *G_t _*grow as *t *decreases. Accordingly, the numbers of edges and vertices of *G_t _*increased monotonically as *t *decreased from 0.02 to 0 in increments of 0.001 (Figure 2). Moreover, the networks *G_t _*had overall more edges and vertices than the corresponding permuted-data networks. Statistically significant differences (*p *≤ 0.01 drawn from permutation testing) in the numbers of edges and vertices present were detected for threshold values satisfying 0.018 ≥ *t *≥ 0.009.

#### Size of the largest connected components

**Figure 3:**
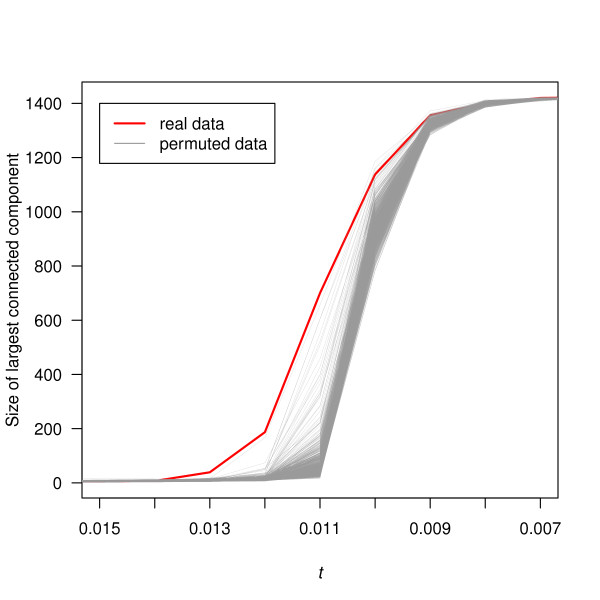
**The size of the largest connected component in the networks with decreasing threshold *t***. The red line represents the real-data network *G_t _*and the gray lines represent the networks of 1,000 permuted data sets. The largest connected components include increasingly more vertices as *t *decreases and eventually include all 1,422 vertices.

Figure 3 shows the size of the largest connected component in the network *G_t _*and in the permuted-data networks as *t *decreased from 0.015 to 0.007. The largest connected component of *G_t _*grew quickly with decreasing *t*. A dominant connected component (larger than any other connected components) emerged at *t *= 0.013 and its growth became considerably steeper subsequently. The largest connected components of the permuted-data graphs, on the other hand, did not start growing before lower values of the threshold were reached, resulting in the major increase in growth happening later than in *G_t_*. Accordingly, their sizes were smaller for most values of the threshold.

One might speculate that those observations were not surprising since, for a fixed value of the threshold *t*, *G_t _*had more edges than did, on average, the graphs constructed from the permuted data (Figure 2). However, networks of more edges and vertices do not necessarily have larger and faster growing connected components. The size of the largest connected component essentially characterizes to which extend the vertices of a network are connected to each other. In fact, even for comparable numbers of edges, the differences in growth between the largest connected components of both *G_t _*and the permuted-data graphs persisted. For example, in the real-data graph, an increase in the number of edges of *G_t _*from 255 to 490, as the threshold decreased from 0.013 to 0.012, was accompanied by an increase in the size of the largest connected component of 148, from 39 to 187. In the permuted-data graphs on the other hand, the size of the largest connected component grew only by 54, from 14 to 68, for an increase in edge number of 335 from 270 to 605 as the threshold decreased from 0.012 to 0.011. Thus, both the size of the largest connected component and the rate at which it grew distinguished the *G_t _*from the networks constructed from the permuted data. Based on above observations, *t *= 0.013 emerged as a threshold of particular interest.

#### Comparison of vertex degree distributions for the threshold 0.013

Table 1 shows the degree distribution of the network *G*_0.013 _and of the 1,000 networks constructed from the permuted data using the same value of *t*. Permuted-data networks had, on average, more vertices with degree one and fewer vertices of higher degrees. In particular, *p*(*d*) for the real-data networks always lay more than one standard deviation away from the mean of *p*(*d*) for the permuted-data networks, except for the three degrees for which the real-data networks had no vertices. This unexpected bias toward high-degree vertices in *G*_0.013 _led us to consider its degree distribution in more detail and to compare it with the degree distributions of other real-data networks obtained by varying *t*.

**Table 1:** Vertex degree distribution of networks for real versus permuted data

*d*		*p*(*d*)
	**Real Data Set**	**Permuted Data Sets (*mean *± *stdev*)**
	
1	0.677	[0.747, 0.831]
2	0.201	[0.119. 0.186]
3	0.0533	[0.0199, 0.0528]
4	0.0345	[0.00184, 0.0210]
5	0.0125	[-0.00168, 0.0124]
6	1.25 × 10^-2^	[-1.85 × 10^-3^, 5.72 × 10^-3^]
7	0	[-1.73 × 10^-3^, 5.81 × 10^-3^]
8	6.27 × 10^-3^	[-1.62 × 10^-3^, 3.41 × 10^-3^]
9	0	[-1.07 × 10^-3^, 1.65 × 10^-3^]
10	0	[-8.54 × 10^-4^, 1.09 × 10^-3^]
11	3.13 × 10^-3^	[-3.14 × 10^-4^, 3.42 × 10^-4^]

The network from the real data has significantly fewer vertices with degree 1 than the networks from the permuted data sets, but more vertices with high degrees.

#### Vertex degree distributions of ${Ĝ}_{t}$

To lessen the risk of including edges likely to exist mostly by chance in *G_t_*, we used ${Ĝ}_{t}$, the subgraph of *G_t _*including only edges with significance *p *≤ 0.01. This changed nothing for *t *= 0.013, as the edges of *G*_0.013 _all had significance *p *≤ 0.001, but resulted in filtering out edges for lower thresholds.

**Figure 4:**
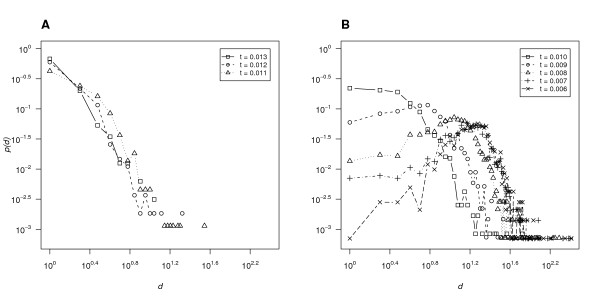
**Vertex degree distributions of networks ${\hat{G}}_{t}$ with *t *ranging from 0.013 to 0.011 (panel A) and from 0.01 to 0.006 (panel B)**. Both axes are on logarithmic scale. Each point represents one vertex degree value. Points are only connected by lines if their degree values differ by 1. **A **Approximately linear curves for 0.013 ≥ *t *≥ 0.011. **B **From 0.01 to 0.006, vertex degree distributions become increasingly bell-shaped.

Figure 4 illustrates part of the vertex degree distributions of the networks ${Ĝ}_{t}$ for 0.013 ≥ *t *≥ 0.006, i.e., only the points (*d*, *p*(*d*)) with *p*(*d*) ≠ 0. Logarithmic scales are used on both axes, so that only points corresponding to nonzero-vertex degrees can be shown. The networks constructed using threshold *t *≥ 0.014 had very few vertices overall and none with degree > 5, and the networks constructed using *t *≤ 0.005 showed very similar patterns to those observed for *t *= 0.006. Therefore, we did not show the degree distributions of these networks.

**Figure 5:**
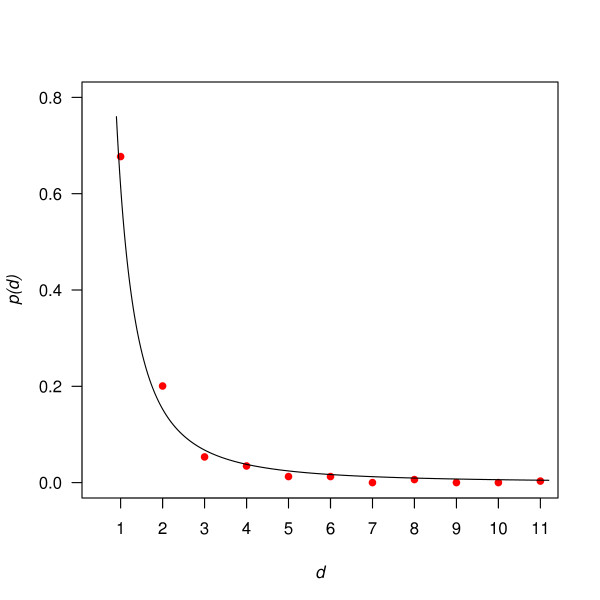
**Vertex degree distribution of network ${\hat{G}}_{0.013}$**. The red points show the observed values and the line in black is the fitting power-law curve of *p*(*d*) = 0.615 × *d*^-2.01^.

The vertex degree distributions of ${Ĝ}_{t}$ with *t *= 0.013, 0.012 and 0.011 were approximately linear (Figure 4-A). Since the scale of Figure 4 is logarithmic, these degree distributions can be approximated by functions of the form *p*(*d*) = *c *× *d*^-*γ *^for suitable positive constants *c *and *γ*. The graphs of such functions are referred to as power curves. We used *least squares *to find the power curves that best fit the points (*d*, *p*(*d*)) for *d *varying from 1 to the highest nonzero-vertex degree of ${Ĝ}_{t}$. The values of *γ *we found for *t *= 0.013, 0.012, and 0.011 were 2.01, 1.73, and 1.3, respectively. However, according to the Kolmogorov-Smirnov test, the resulting functions *t *the degree distributions of ${Ĝ}_{0.012}$ and ${Ĝ}_{0.011}$ very poorly: for both networks, the null hypothesis that the observed degree distribution follows the best-fitting power curve was rejected with *p *< 0.0005. For ${Ĝ}_{0.013}$ on the other hand, the corresponding *p *value was 0.366, suggesting that the null hypothesis was still plausible. Figure 5 shows the degree distribution of ${Ĝ}_{0.013}$ and the fitting power curve for *p*(*d*) = 0.615 × d^-2.01^.

The vertex degree distributions of ${Ĝ}_{t}$ became increasingly bell-shaped as *t *decreased from 0.010 to 0.006 (Figure 4-B). This occurred as more edges of low weight were likely to be included in ${Ĝ}_{t}$ due to chance rather than to biological significance and ${Ĝ}_{t}$ therefore progressively resembled random networks. The vertex degree distributions of such random networks follow a Poisson distribution $p\left(d\right)=\frac{\lambda^{d}}{d!}e^{-\lambda }$, where *λ *is the mean, in our case the average vertex degree (see Additional file 1 for the Poisson distribution fitting curves for each cutoff *t*).

A vertex degree distribution can still follow a Poisson distribution even if it is not bell-shaped, which happens when *λ *is small. For ${Ĝ}_{0.013}$, $\lambda =\frac{2\times 255}{1,422}\approx 0.366$, where 255 was the number of edges in ${Ĝ}_{0.013}$ and 1,422 was the total number of SNPs. For such a small value of *λ*, a Poisson distribution is not bell-shaped. Hence, ruling out the possibility that ${Ĝ}_{0.013}$ follows a Poisson degree distribution required further investigation.

We therefore tested the hypothesis that the vertex degrees of ${Ĝ}_{0.013}$ followed a Poisson distribution. The construction process of the networks ${Ĝ}_{t}$ can be described as attaching edges to 1,422 vertices and then removing the vertices of degree zero. If this attachment were random, and no degree-zero vertices were removed, the vertex degrees would follow a Poisson distribution. When degree-zero vertices are removed, as was the case here, the theoretical Poisson distribution has to be adjusted as follows:

(5) $$P_{0}\left(d\right)=\left\{\begin{array}{c} \frac{\lambda^{d}}{kd!}e^{-\lambda }\;\;\text{if}\;\;d\geq 1\\ P_{0}\left(0\right)=0 \end{array}\right)$$

where *k *= 1 - *P *(0) = 1 -e^-*λ *^normalized the adjusted distribution *P*_0_(*d*) since *P*_0_(0) = 0. According to the Kolmogorov-Smirnov test, the null hypothesis that the vertex degrees of ${Ĝ}_{0.013}$ were drawn from the adjusted Poisson distribution *P*_0_(*d*) or, equivalently, that its edge attachment was random was rejected with *p *= 0.001.

Networks with degree distributions of the form *p*(*d*) = *c *× *d*^-*λ *^are said to have power-law distributions and are often called scale-free in the literature [30]. Although the term is usually only applied to very large networks, at least two to three magnitudes larger than those considered here, our results nevertheless suggest that the network *G*_0.013 _was scale-free, or at least approximately so.

#### Network G_0.013_

**Figure 6:**
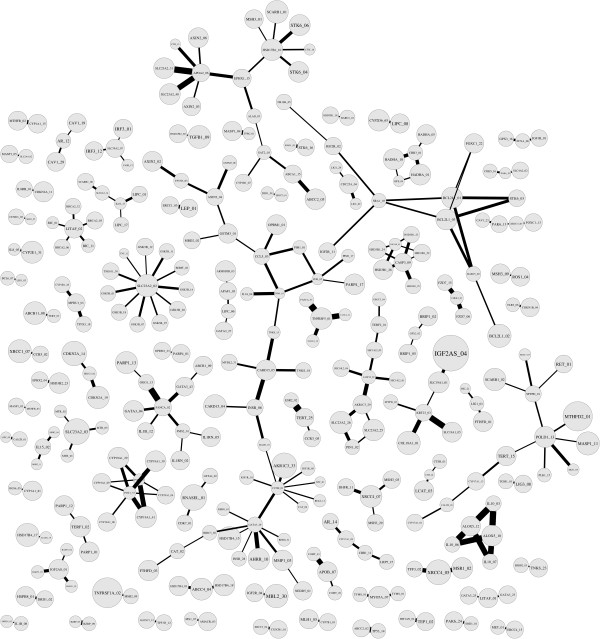
**Statistical epistasis network ${\hat{G}}_{0.013}$**. There are 319 vertices and 255 edges. The network has 79 connected components and the largest one has 39 vertices. The width of an edge and the size of a vertex are in proportion to their weights. The length of an edge is for layout purposes only. The graph is rendered by the software Graphviz.

The network *G*_0.013 _(Figure 6) had 255 edges, 319 vertices, and 79 connected components (see Additional files 2, 3, 4 for subdivided graphs with only the largest component, other relatively large components, and the rest small components). All of those 255 edges have significance *p *≤ 0.001. This could be partially explained by the fact that these top 255 edges had relatively high weights and thus more likely obtained smaller p-values using permutation testing. The largest connected component had 39 vertices. This was more than twice as large as the second largest connected component of size 18. In Figure 6, the size of a vertex is proportional to the main effect of the corresponding SNP and the width of an edge is proportional to the strength of the interaction between the two SNPs it joins (see Additional files 5 and 6 for the standard network vertex and edge files). The network provides a clear visualization of the pairs of SNPs which had the strongest synergetic effect on bladder cancer, as well as the strength of these effects and of the individual SNPs involved in the strongest interactions. Most importantly, the network shows which synergetic pairs shared a SNP, and thereby captures the entire pairwise interaction space.

As is the case for biological pathways, this statistical epistasis network showed very few cycles. In particular, there were no connected triangles. That is, vertices did not interact with their neighbors' neighbors. Moreover, in accordance with its power-law degree distribution, the network had a few vertices with degrees that were much higher than the average, while the majority of vertices connected directly to only one other vertex. Finally, vertices with high degrees or connected with wide edges were not necessarily of large size (see Additional file 7 for the linear regression showing no correlation between vertex size and vertex connection).

## Discussion

The goal of this study was to infer and characterize statistical epistasis networks in a large population-based study of bladder cancer susceptibility. We observed distinguishing topologies of the networks assembled using the cancer data and the implication that a group of SNPs may jointly modify the disease outcome. Specifically, the networks *G_t _*had many more high-degree vertices and their largest connected components emerged earlier and grew faster than expected. These characteristics were the most apparent when *t *= 0.013. The network *G*_0.013 _was shown to be approximately scale-free, an important property found in various natural and social networks. This property was no longer observable when *t *further decreased and edges representing weaker and possibly less biologically relevant pairwise interactions were added.

The network *G*_0.013 _allows for some interesting observations about the structure of the pairwise interaction space of the genetic data. First, SNPs aggregate to form connected components, which may indicate that multiple SNPs jointly modify disease outcome. In *G*_0.013_, SNPs are grouped into 79 connected components of size ranging from 2 to 39. These connected components show various structural patterns, also known as *motifs*, including lines, crosses, and stars. The largest connected component has a tree-like structure. This may imply the existence of unique interaction patterns among groups of SNPs.

Second, the network has an approximately scale-free topology and an ensemble of particularly high-degree vertices, which suggests that it may be exceptionally robust. Scale-free networks permeate natural and social sciences [47-49]. The most well-known scale-free networks are the backbone of the Internet and social networks. In biology, scale-free topologies have been found in metabolic networks [31], protein-protein interaction networks [33], and gene-regulatory networks [34]. Those various scale-free networks share an intriguing property: the value of *γ *in the degree distributions *p*(*d*) = *c *× *d *^-*γ *^mostly satisfies 2 ≤ *γ *≤ 3 [47], which is also the case for *G*_0.013 _(*γ *= 2.01). As more scale-free networks are being discovered in a variety of fields, a question remains: how can systems as fundamentally different as the cell and the Internet have a similar architecture and obey the same laws [47]? Scale-free networks typically have many vertices with low degrees and a few vertices with high degrees, also known as *hubs *[30]. This essentially differentiates scale-free networks from random networks where the majority of vertices have average degrees. The probability *p*(*d*) of degree *d *in the Poisson distribution decreases exponentially as *d *increases, and thus random networks are very unlikely to have hubs with degrees much larger than the average. The existence of hubs in a scale-free network implies strong robustness against failures. Because random vertex removal is very unlikely to affect hubs, the connectivity of the network most likely remains intact. In biological networks, this robustness translates into the resilience of organisms to intrinsic and environmental perturbations. For instance, in protein-protein interaction networks [33], most proteins interact with only one or two other proteins but a few are able to interact to a large number. Such hub proteins are rarely affected by mutations and organisms can remain functional under most perturbations. The simultaneous emergence of scale-free topologies in many biological networks suggests that evolution has favored such a structure in natural systems. Moreover, it suggests that the robustness of natural systems does not only result from inherent genetic redundancy but also, and maybe more importantly, from the topological organization of entities and interactions [33]. Although our epistasis network is developed based on statistical rather than on real bio-chemical interactions, it is interesting to observe similar topologies between biological and statistical networks.

Third, the existence of main effects does not necessarily correlate with the occurrence of interactions. This, in turn, suggests that many current main-effect-prioritized methods might have overlooked SNPs contributing to the disease susceptibility through their interactions with other SNPs rather than through their main effects. As shown in the graph, large main-effect SNPs do not necessarily associate with strong pairwise interactions or interact with many other SNPs. Instead, SNPs involved in potential important pairwise interactions, such as those located on the central path of the largest connected component, often have relatively small main effects.

The statistical epistasis network approach has many advantages. 1) Networks allow for efficiently visualizing both main and epistatic effects and how they interplay. 2) Networks serve as a very intuitive tool to study pairwise interactions and to characterize the entire epistatic interaction space. Moreover, they may also help identify higher-order interactions by grouping SNPs into connected components. High-order epistasis does not necessarily require detectable pairwise interactions between SNPs. However, given that current computational power allows only for exhaustively enumerating pairwise interactions in moderate-size data sets, pairwise interaction networks may serve as a useful guide to explore higher-order epistasis among SNPs that exhibit lower-order interactions. 3) Our network model is assembled using the entire set of available SNPs without limiting ourselves to only high main-effect ones. This reduces the risk of overlooking candidate SNPs that are involved in important interactions but with low main effects. 4) Network topological analyses are used to systematically determine the best network that captures the genetic architecture of a data set. 5) Networks, along with graph theory, are well-developed fields, and many advanced techniques and analytical tools are likely to benefit future network-based epistasis studies. In particular, additional topological properties such as motif distribution and network diameter [30,42] are worth investigating.

Among the limitations of this approach is that it is still under development and no user-friendly interface is available yet. Different data sets may require different analytical tools and a fully automated analysis software may therefore not be appropriate. Moreover, since the approach aims at highlighting pairs of SNPs with strong pairwise interactions, it is likely to overlook SNPs that are only involved in higher-order interactions. As mentioned previously, strong three- or higher-order interactions may exist despite the absence of pairwise interactions.

The statistical epistasis network approach we used can be extended in the following ways. 1) The network *G*_0.013 _will be further studied for bladder cancer association. Through a closer investigation, such as gene ontologies and biological pathways, on those 319 SNPs in the network, especially those 39 SNPs in the largest connected component, we expect to prioritize gene categories with high bladder cancer susceptibility, and to testify whether SNP interactions tend to happen within the same category or across categories. Other possible applications include using the network to train classifiers in predicting bladder cancer risk [50] and to supervise data mining methods for identifying high-order genetic interactions [27]. 2) The approach can also be applied to other data sets. We are particularly interested in investigating network topologies in larger data sets or data associated with other diseases. 3) To corroborate the present results, future studies could use metrics other than information theoretical measures, such as SNP and gene annotation or SURF scores, which are obtained by directly assessing genetic variants depending on their phenotype relevance using machine learning techniques [51]. 4) Given the effect of smoking [37] and arsenic exposure [41,52] on bladder cancer prevalence, an additional next step is to account for gene-environment interactions in our analyses. This can be achieved by adding these environmental factors to our model, and investigating how the environmental background on which the genes are expressed modify the conclusions we draw.

## Conclusions

In this study, we proposed a statistical epistasis network approach that is able to capture the global landscape of gene-gene interactions in a large population-based bladder cancer data set. Through an exhaustive enumeration of all possible pairwise interactions and network topological analyses, a distinctive network is systematically identified which shows unique properties. It has a significantly large connected component and an intriguing approximate scale-free topology that permeate natural and technical networks. Specifically in the context of biological networks, scale-free is well recognized as an outcome interaction topology of robust organisms resulted by natural evolution. The observation of such a network topology in the bladder cancer data set may indicate a global interactive structure embedded in the genetic architecture of bladder cancer.

The derived network from this study may further benefit bladder cancer studies through closer examinations of SNP characteristics. In addition to a global interaction picture of bladder cancer depicted by this network, further studies on individual gene ontology and biological pathway categorization may provide important insight on prioritizing inter- or intra-category genetic interactions. Moreover, the proposed network approach holds the promise characterizing a broader gene-gene interaction landscape in epistasis studies, and is expected to be applied to other data sets, especially large-scale ones.

## Competing interests

The authors declare that they have no competing interests.

## Authors' contributions

TH designed the study, performed the analyses, and drafted the manuscript. NASA participated in the design of the study and performed the analyses. JWK participated in the analyses and helped to draft the manuscript. ASA and MRK carried out the data collection and the genotyping, and helped to draft the manuscript. JHM conceived of the study, and participated in its design and coordination and helped to draft the manuscript. All authors read and approved the final manuscript.

## Supplementary Material

Additional file 1 **Poisson vertex degree distribution fitting curves of networks ${\hat{G}}_{t}$ with *t *ranging from 0.013 to 0.011 (panel A) and from 0.01 to 0.006 (panel B)**. If networks ${Ĝ}_{t}$ were built through the process of randomly linking two vertices and then removing degree-zero vertices, their vertex degrees would follow an adjusted Poisson distribution $P_{0}\left(d\right)=\frac{\lambda^{d}}{kd!}e^{-\lambda }$, *d *> 0, where the normalizing factor *k *= *P *(0) = 1 - *e*^-*λ *^and *λ *is the average vertex degree of networks ${Ĝ}_{t}$. Both axes are on logarithmic scale. Click here for file

Additional file 2 **The largest connected component in network ${\hat{G}}_{0.013}$**. There are 39 SNPs connected in the largest component. Click here for file

Additional file 3 **Other large connected components in network ${\hat{G}}_{0.013}$**. The sizes of the other large connected components are ranging from 5 to 18. Click here for file

Additional file 4 **Small connected components in network ${\hat{G}}_{0.013}$**. The small connected components only have 2 to 4 SNPs. Click here for file

Additional file 5 **Standard network vertex file of ${\hat{G}}_{0.013}$**. The file shows a list of vertices and their weights. Click here for file

Additional file 6 **Standard network edge file of ${\hat{G}}_{0.013}$**. The file shows a list of edges and their weights. Click here for file

Additional file 7 **Vertex main effect as a function of degree (panel A) and the total weight of attached edges (panel B) in network ${\hat{G}}_{0.013}$**. The vertex main effect is independent of its degree and summed weight of all attached edges. Lines show the correlations using linear regression. Click here for file
