# Supplementary figures and images for: Characterizing genetic interactions in human disease association studies using statistical epistasis networks
Source: BMC Bioinformatics. 2011 Sep 12;12:364. doi: 10.1186/1471-2105-12-364 (PMC3215301; doi:10.1186/1471-2105-12-364)

**A**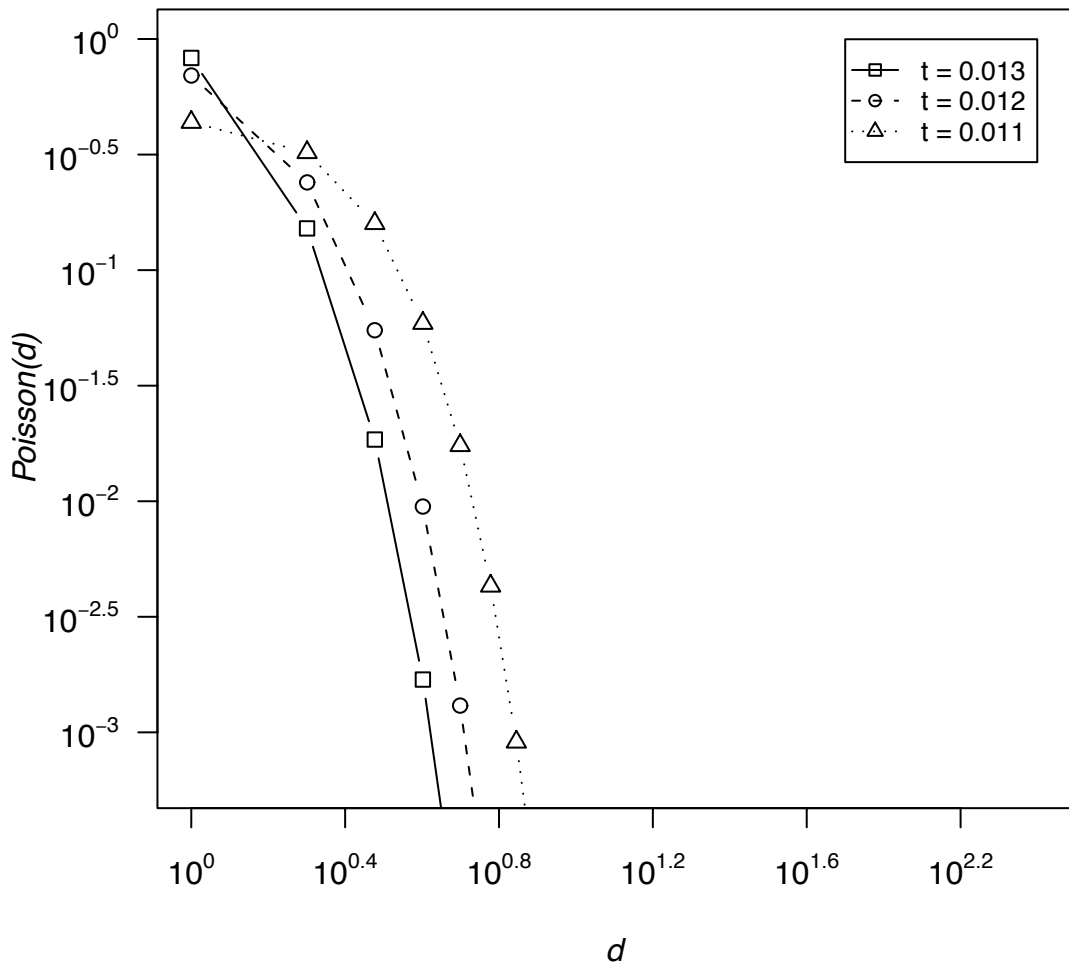**B**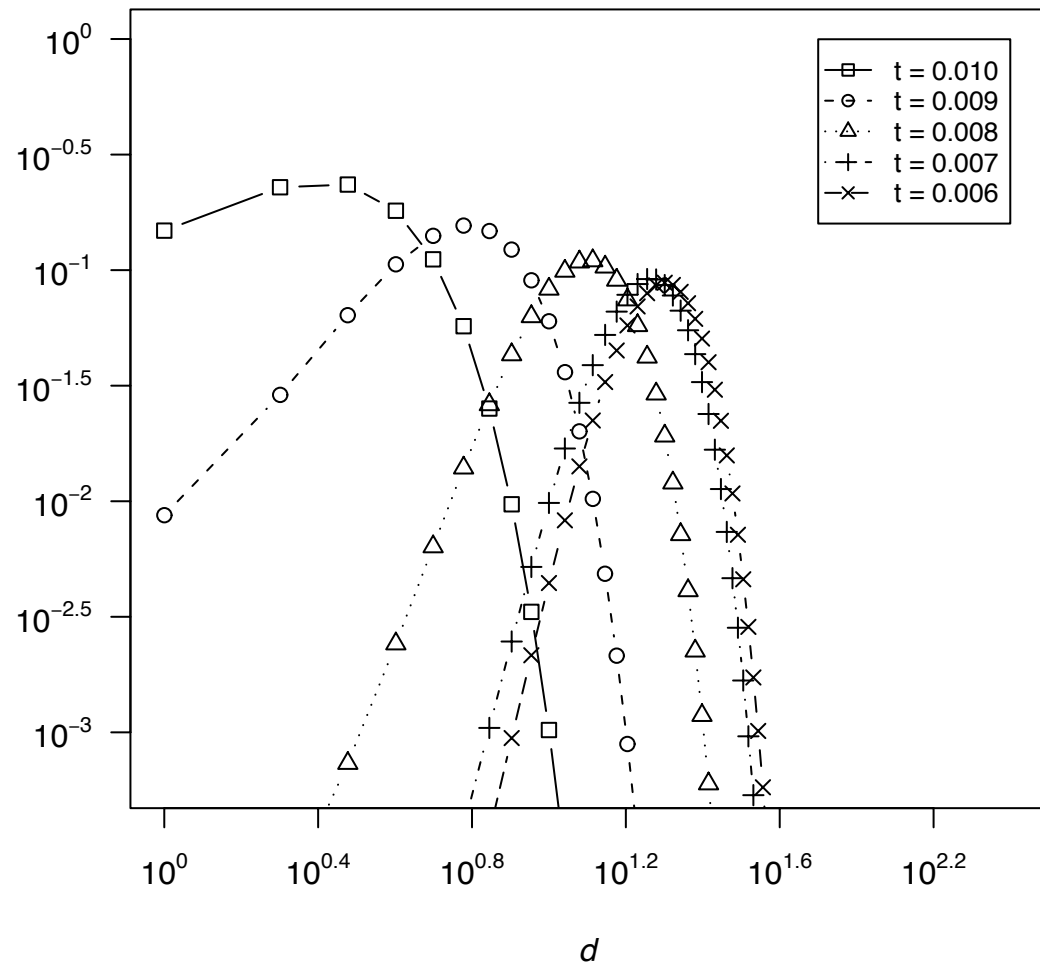

Supplement: Additional file 1 — Poisson vertex degree distribution fitting curves of networks G^t with t ranging from 0.013 to 0.011 (panel A) and from 0.01 to 0.006 (panel B). If networks Ĝt were built through the process of randomly linking two vertices and then removing degree-zero vertices, their vertex degrees would follow an adjusted Poisson distribution P0(d)=λdkd!e-λ, d > 0, where the normalizing factor k = P (0) = 1 - e-λ and λ is the average vertex degree of networks Ĝt. Both axes are on logarithmic scale. [file 1471-2105-12-364-S1.PDF]

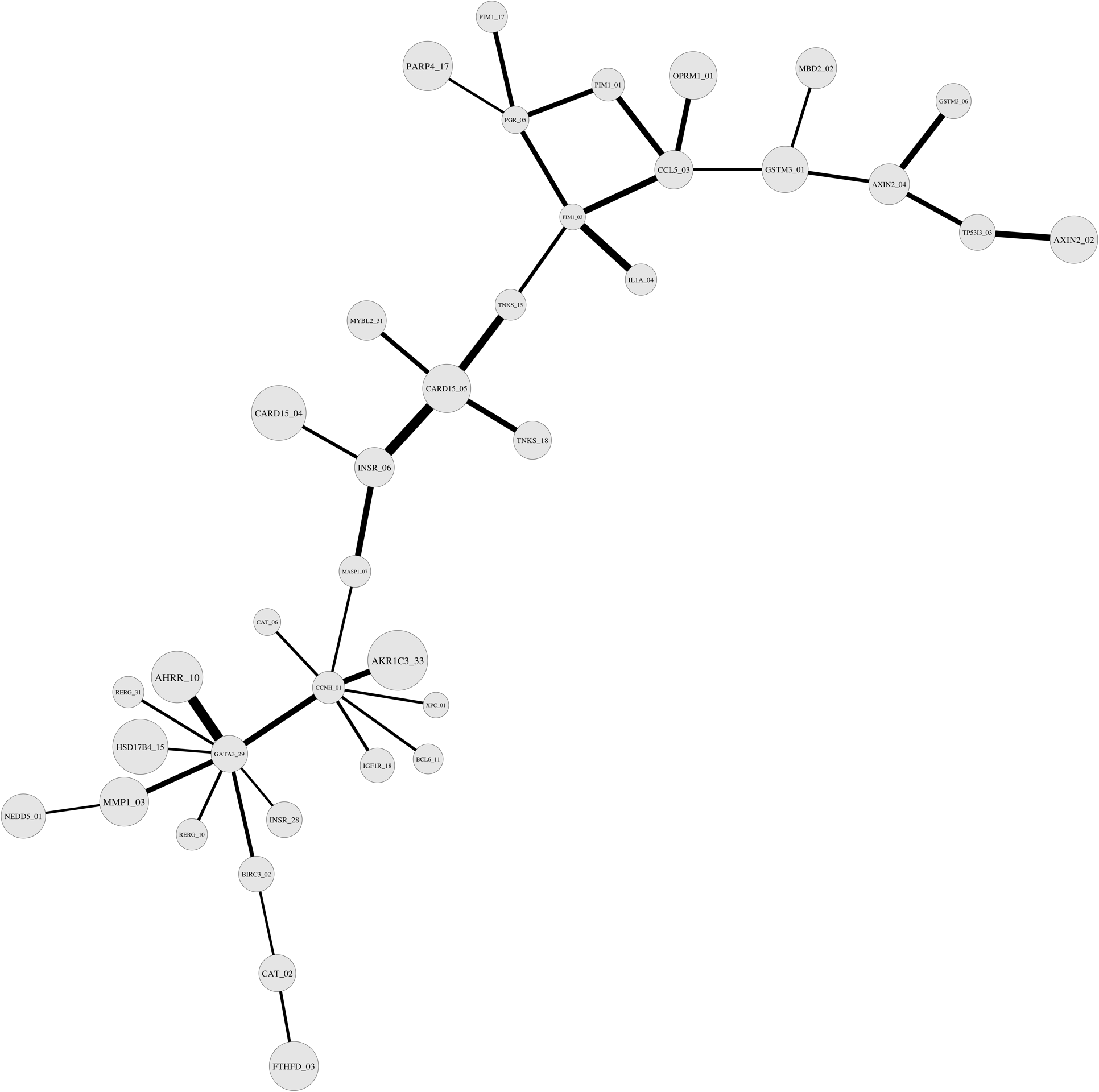

Supplement: Additional file 2 — The largest connected component in network G^0.013. There are 39 SNPs connected in the largest component. [file 1471-2105-12-364-S2.PDF]

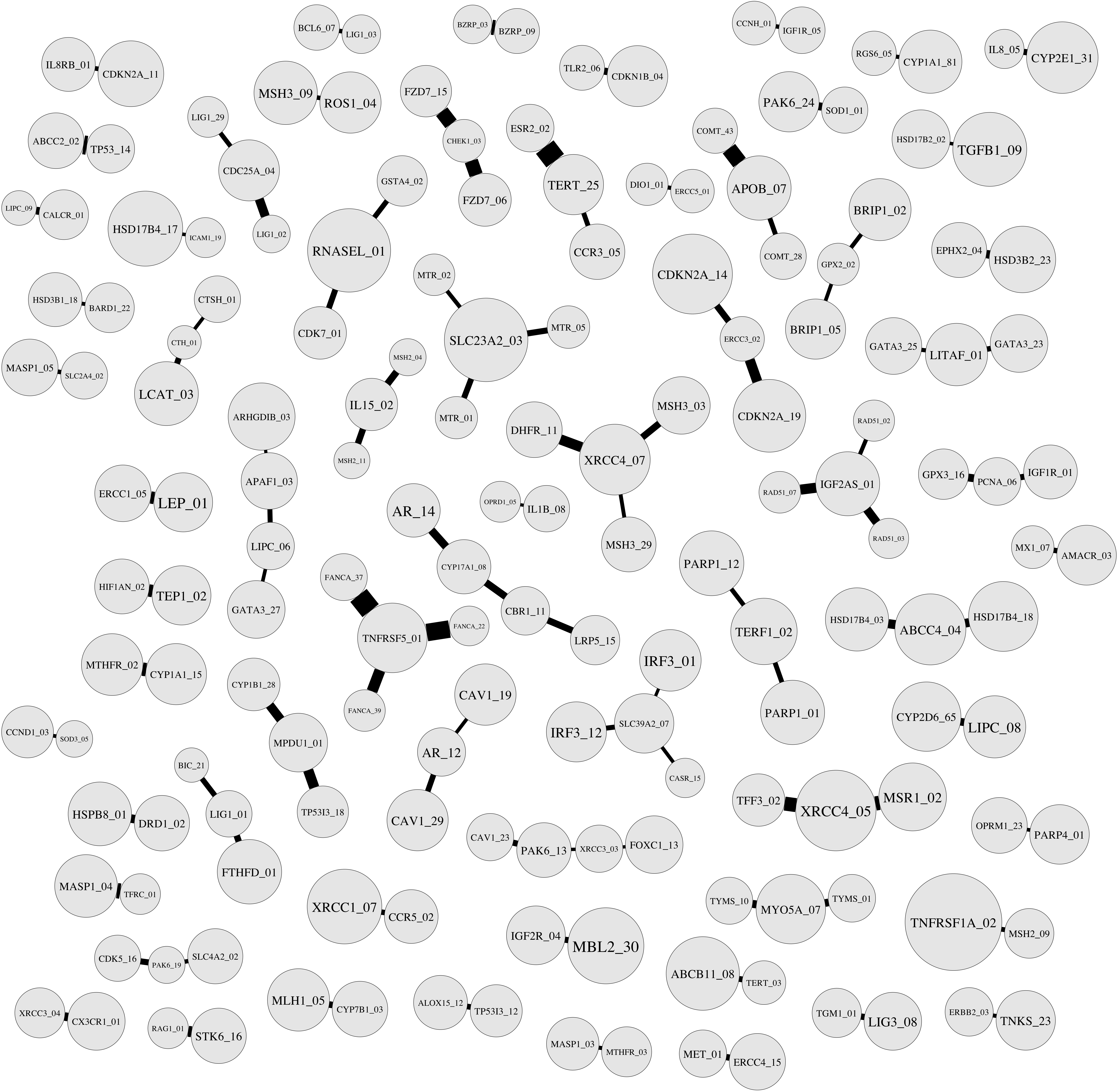

Supplement: Additional file 4 — Small connected components in network G^0.013. The small connected components only have 2 to 4 SNPs. [file 1471-2105-12-364-S4.PDF]

**A**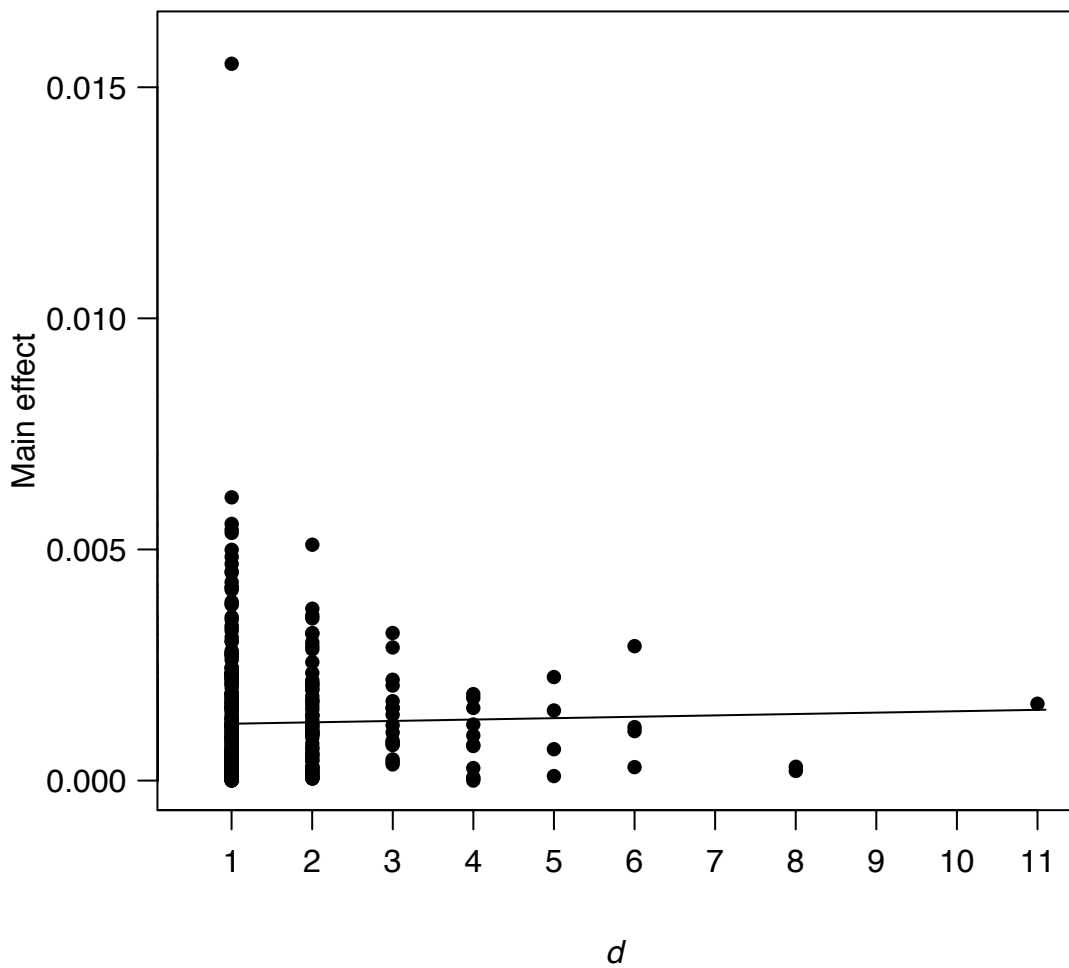**B**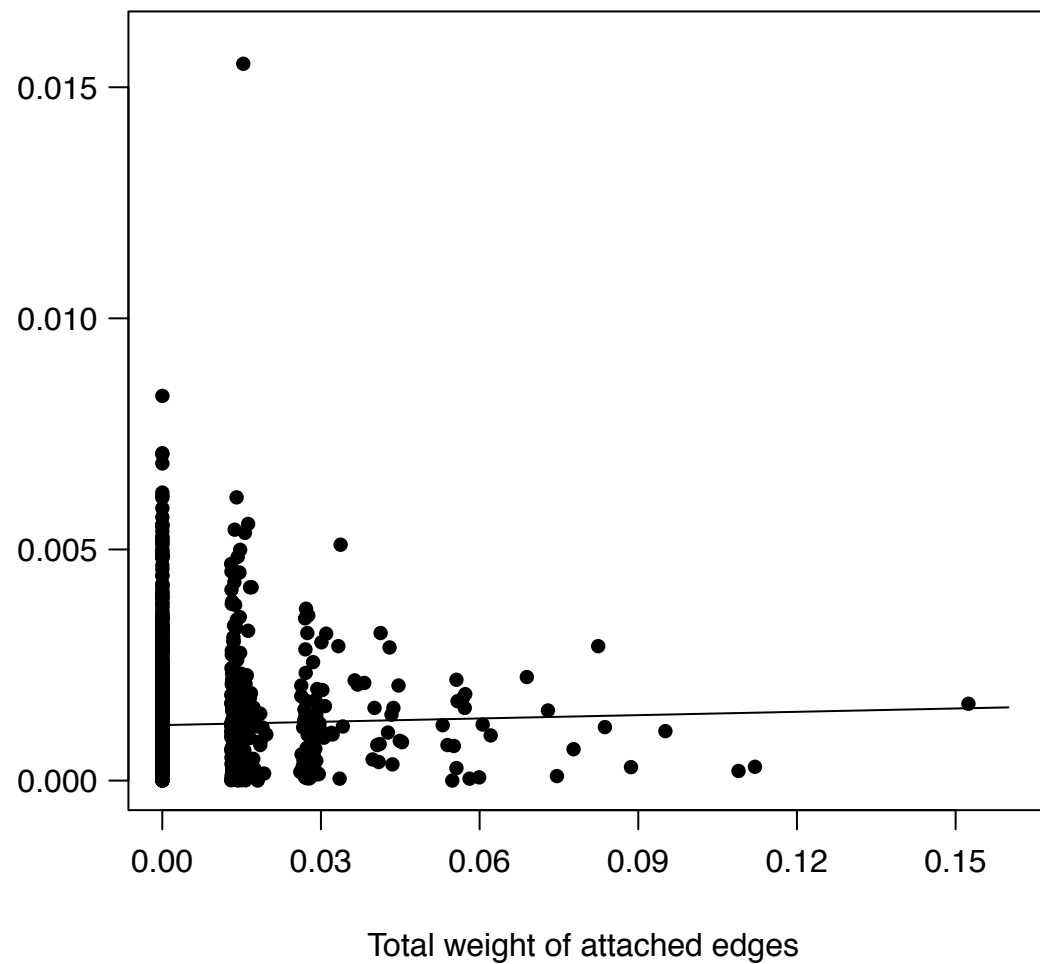

Supplement: Additional file 7 — Vertex main effect as a function of degree (panel A) and the total weight of attached edges (panel B) in network G^0.013. The vertex main effect is independent of its degree and summed weight of all attached edges. Lines show the correlations using linear regression. [file 1471-2105-12-364-S7.PDF]
