# Supplementary material for: Characterizing genetic interactions in human disease association studies using statistical epistasis networks
Source: BMC Bioinformatics. 2011 Sep 12;12:364. doi: 10.1186/1471-2105-12-364 (PMC3215301; doi:10.1186/1471-2105-12-364)
Supplement: Additional file 3 — Other large connected components in network G^0.013. The sizes of the other large connected components are ranging from 5 to 18. [file 1471-2105-12-364-S3.PDF]

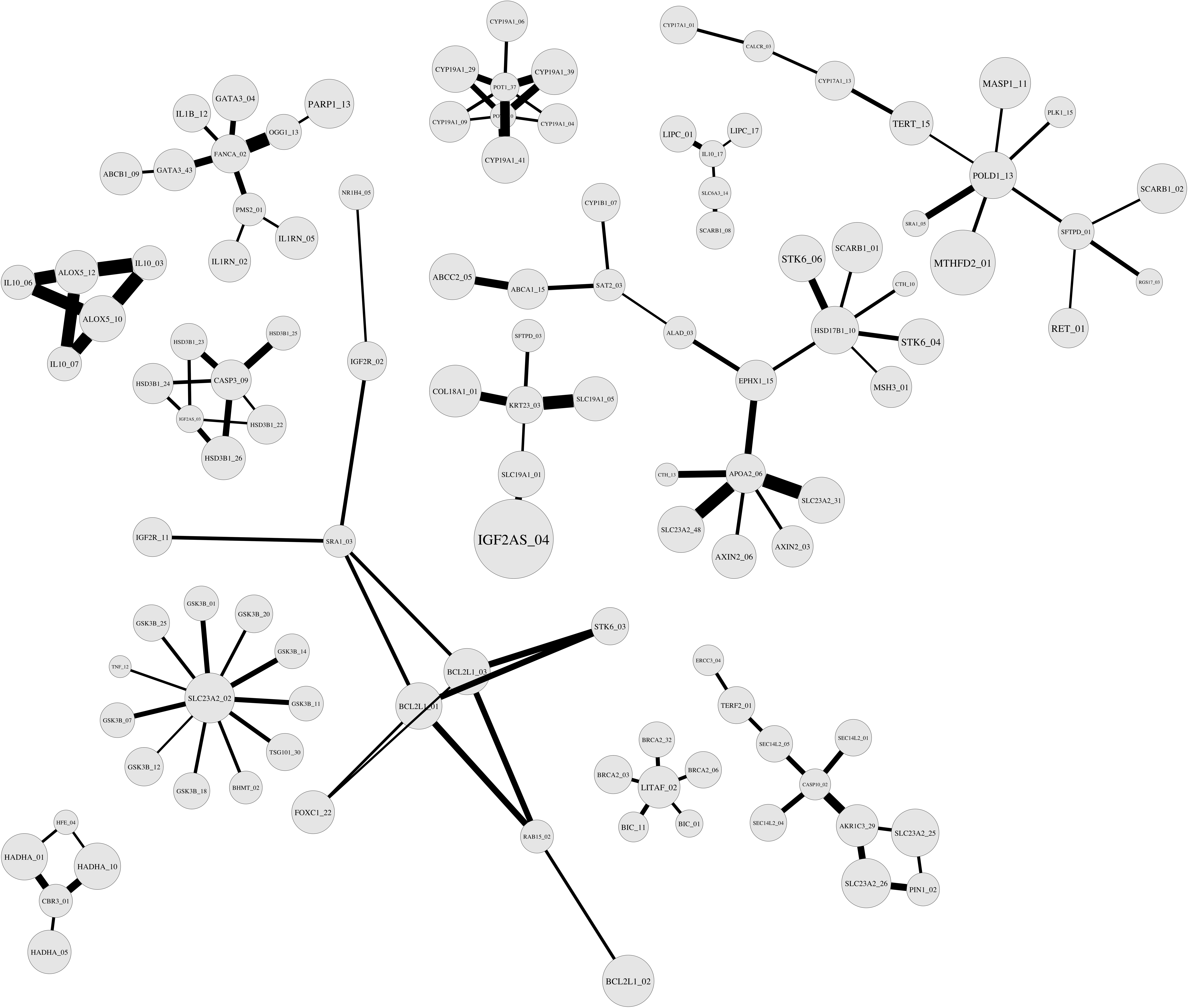

CYP19A1_06
CYP19A1_29
POT1_37
CYP19A1_39
CYP19A1_09
CYP19A1_04
CYP19A1_41
CYP17A1_01
CALCR_03
CYP17A1_13
TERT_15
MASP1_11
PLK1_15
POLD1_13
SRA1_05
MTHFD2_01
SCARB1_02
SFTPD_01
RGS17_03
RET_01
LIPC_01
LIPC_17
IL10_17
SLC6A3_14
SCARB1_08
IL1B_12
GATA3_04
PARP1_13
OGG1_13
FANCA_02
ABCB1_09
GATA3_43
PMS2_01
IL1RN_05
IL1RN_02
IL10_06
ALOX5_12
IL10_03
ALOX5_10
IL10_07
HSD3B1_23
HSD3B1_25
CASP3_09
HSD3B1_24
IGF2AS_03
HSD3B1_22
HSD3B1_26
NR1H4_05
IGF2R_02
CYP1B1_07
ABCC2_05
ABCA1_15
SAT2_03
ALAD_03
STK6_06
SCARB1_01
CTH_10
HSD17B1_10
STK6_04
MSH3_01
EPHX1_15
SFTPD_03
COL18A1_01
KRT23_03
SLC19A1_05
SLC19A1_01
IGF2AS_04
CTH_13
APOA2_06
SLC23A2_31
SLC23A2_48
AXIN2_06
AXIN2_03
IGF2R_11
SRA1_03
GSK3B_01
GSK3B_20
GSK3B_25
GSK3B_14
TNF_12
SLC23A2_02
GSK3B_11
GSK3B_07
GSK3B_12
TSG101_30
GSK3B_18
BHMT_02
STK6_03
BCL2L1_03
BCL2L1_01
FOXC1_22
RAB15_02
BCL2L1_02
ERCC3_04
TERF2_01
SEC14L2_05
SEC14L2_01
CASP10_02
SEC14L2_04
AKR1C3_29
SLC23A2_25
SLC23A2_26
PIN1_02
BRCA2_32
BRCA2_03
BRCA2_06
LITAF_02
BIC_11
BIC_01
HFE_04
HADHA_01
HADHA_10
CBR3_01
HADHA_05
